# Screening for Biomarkers for Progression from Oral Leukoplakia to Oral Squamous Cell Carcinoma and Evaluation of Diagnostic Efficacy by Multiple Machine Learning Algorithms

**DOI:** 10.3390/cancers14235808

**Published:** 2022-11-25

**Authors:** Fengyang Jing, Jianyun Zhang, Xinjia Cai, Xuan Zhou, Jiaying Bai, Heyu Zhang, Tiejun Li

**Affiliations:** 1 Department of Oral Pathology, Peking University School and Hospital of Stomatology, National Center of Stomatology, National Clinical Research Center for Oral Diseases, National Engineering Laboratory for Digital and Material Technology of Stomatology, Beijing Key Laboratory of Digital Stomatology, Research Center of Engineering and Technology for Computerized Dentistry Ministry of Health, NMPA Key Laboratory for Dental Materials, Beijing 100081, China; 2 Research Unit of Precision Pathologic Diagnosis in Tumors of the Oral and Maxillofacial Regions, Chinese Academy of Medical Sciences (2019RU034), Beijing 100081, China; 3 Central Laboratory, Peking University School and Hospital of Stomatology, Beijing 100081, China

**Keywords:** oral leukoplakia, oral squamous cell carcinoma, machine learning, diagnostic model, immune infiltration

## Abstract

**Simple Summary:**

The study was the first to screen seven genes and identify the set of genes using twelve machine learning algorithms that predict the progression from oral leukoplakia to oral squamous cell carcinoma. We verified these genes by RT-qPCR experiments and speculated on the possible molecular mechanisms through the results. These genes could be used as biomarkers for early diagnosis and predicting patients with a high risk of malignant transformation. This will aid in early intervention, improve the patient prognosis, and reduce the incidences of oral squamous cell carcinoma.

**Abstract:**

The aim of the study is to identify key genes during the progression from oral leukoplakia (OL) to oral squamous cell carcinoma (OSCC) and predict effective diagnoses. Weighted gene co-expression network analysis (WGCNA) and differential expression analysis were performed to identify seven genes associated with the progression from OL to OSCC. Twelve machine learning algorithms including k-nearest neighbor (KNN), neural network (NNet), and extreme gradient boosting (XGBoost) were used to construct multi-gene models, which revealed that each model had good diagnostic efficacy. The functional mechanism or the pathways associated with these genes were evaluated using enrichment analysis, subtype clustering, and immune infiltration analysis. The enrichment analysis revealed that the genes enriched were associated with the cell cycle, cell division, and intracellular energy metabolism. The immunoassay results revealed that the genes primarily affected the infiltration of proliferating T cells and macrophage polarization. Finally, a nomogram and Kaplan–Meier survival analysis were used to predict the prognostic efficacy of key genes in OSCC patients. The results showed that genes could predict the prognosis of the patients, and patients in the high-risk group had a poor prognosis. Our study identified that the seven key genes, including *DHX9, BCL2L12, RAD51, MELK, CDC6, ANLN,* and *KIF4A,* were associated with the progression from OL to OSCC. These genes had good diagnostic efficacy and could be used as potential biomarkers for the prognosis of OSCC patients.

## 1. Introduction

Oral squamous cell carcinoma (OSCC) is the most common type of oral malignancy, and the five-year overall survival values for the whole cohort, oral cavity, oropharynx, hypopharynx, and larynx are 24.1%, 25.91%, 19.2%, 13.4%, and 38.0%, respectively [1,2,3,4]. Patients with OSCC have poor prognoses. However, early diagnosis and treatment can help to improve survival and prevent recurrences of cancer [5,6], thereby improving the prognosis of patients with OSCC. Oral potential malignant diseases (OPMDs), including leukoplakia, erythema, lichen planus, and niacin stomatitis, are precancerous lesions with different malignant transforming abilities to progress to OSCC [7,8,9]. Oral leukoplakia (OL) is the most common OPMD, with a 1.1–40.8% malignant transformation rate [10,11]. A recent study showed that out of 5000 patients with OL, approximately one-third of the patients would eventually develop OSCC, and most patients developed OSCC within a year of OL diagnosis [11]. If the risk of OPMD transformation could be predicted, it would aid in the early diagnosis and treatment of the patients. Further, systemic chemoprophylaxis could be used to treat patients with a high risk of progression to OSCC. This will help reduce the incidence of oral cancer.

Currently, the diagnosis of leukoplakia is based on clinical features and histopathological evaluations by pathologists. This may differ based on their experience and could lead to discrepancies in results. The lack of standardized evaluation criteria may prevent early diagnosis and treatment of patients to prevent OSCC [12,13,14]. Various studies have identified genes that may predict the progression from OL to OSCC, of which *ATM* is the most extensively studied gene, which plays an important role in the cell cycle [15]. Previous studies have investigated the role and methylation pattern of DNA mismatch repair (MMR) genes such as *hMLH1* and *hMSH2* in oral carcinogenesis and their association with various oral malignancies [16,17]. Multiple studies have used different strategies to predict the risk of progression from OL to oral cancer; however, no study has identified biomarkers that can effectively predict the risk of progression from OL to oral cancer.

We believe that a specific gene or a class of genes cannot effectively predict the risk of progression from OL to OSCC. Hence, it is necessary to identify genes using various algorithms. This is now possible due to advancements in bioinformatics and machine learning. Previous studies have focused on the role of a specific class of genes, such as MMR genes, in the progression from OL to OSCC. In the current study, we attempted to screen for key genes associated with the progression from OL and OSCC. Various models were constructed using twelve machine learning algorithms to evaluate the diagnostic efficacy of the key genes in predicting the progression from OL to OSCC. Further, the impact of these key genes on the prognosis of OSCC patients was also evaluated. Our results revealed that despite the differences, all the models had good diagnostic efficacy, indicating that these key genes may be involved in the progression from OL to OSCC. Further, these key genes could also affect the prognosis of OSCC patients. Our results revealed key diagnostic genes that could predict the progression from OL to OSCC. This will allow for early diagnosis and treatment of high-risk OL patients and help reduce the incidences of OSCC.

## 2. Materials and Methods

### 2.1. Cell Cultures

DOK cells and SCC-15 cells were acquired from Central Laboratory of Peking University. Standard cell culture techniques were followed while culturing these cells. DOK cells were grown in Dulbecco’s modified Eagle’s medium supplemented with 10% fetal bovine serum. SCC-15 cells were grown in a 1:1 mixture of Dulbecco’s modified Eagle’s medium/Ham’s F12 medium supplemented with 400 ng/mL hydrocortisone and 10% fetal bovine serum. The cells were grown in an incubator at 37 °C and 5% CO_2_.

### 2.2. Publicly Available Data Collection and Processing

OL and oral cancer-related datasets including GSE26549 [18], GSE85195 [19], and GSE85514 [19] were retrieved from the Gene Expression Omnibus (GEO) database via the GEOquery package [20]. The species set for data collection was *Homo sapiens*. The sequencing platform for GSE26549 was GPL6244, which was comprised of 86 OL samples, including 54 hyperplasia and 32 dysplasia samples. In 35 patients, OL eventually progressed to OSCC. The sequencing platform GPL6480 was used for GSE85195, which included 15 OL and 34 OSCC samples. The sequencing platform for GSE85514 was GPL22311, which included 24 OL and 38 OSCC samples (Supplementary Table S1). The data were normalized using the “linear models for microarray data (limma)” R package.

The “TCGAbiolinks” R package was used to retrieve the OSCC dataset from The Cancer Genome Atlas (TCGA) database [21]. TCGA-OSCC comprised 361 samples; 329 were tumor tissue samples (tumor), and 32 were adjacent normal tissue samples (normal). The data were in TPM format.

The somatic mutation data of OSCC patients (n = 361) were obtained from TCGA-GDC (https://portal.gdc.cancer.gov/) (accessed on 28 September 2022) by choosing “Masked Somatic Mutation.” The data were preprocessed using the VarScan software and visualized by the “maftools” R package.

Additionally, the matched clinical data of TCGA-OSCC patients, including age, survival status, and follow-up status, were obtained from TCGA-GDC. The patients with no information about their survival and incomplete TNM staging were excluded from the analysis. Finally, a total of 361 patient data was used for subsequent analysis.

### 2.3. Weighted Gene Co-Expression Network Analysis (WGCNA)

WGCNA [22] allows the identification of co-expressed gene modules and explores the relationship between gene networks and phenotypes to study the core genes in the network. The soft threshold was calculated by the pickSoftTreshold function, followed by a scale-free network based on the soft threshold to build a topological matrix and perform hierarchical clustering. The data were randomly divided into internal training and validation sets in a 1:1 ratio for preservation analysis. The number of replacement tests was 200 times. The modules with *z*-score < 2 were removed after z-score extraction. The correlation between modules and clinical features was analyzed by Pearson’s correlation coefficient, and the module genes with the highest correlation were selected for subsequent analysis.

### 2.4. Differential Expression Analysis

The samples from the GSE85195 dataset were divided into the OL and the OSCC groups, with 15 samples in the OL group and 32 samples in the OSCC group. The differential expression analysis in the different groups was performed using the “limma” R package. The genes with adjusted *p* < 0.05 and |log fold change (FC)| > 1 were identified as differential expression genes, of which genes with |logFC| > 1 were considered up-regulated in the OSCC group compared to the OL group. The genes with |logFC| < −1 were deemed down-regulated in the OSCC group compared to the OL group. The differential analysis results were illustrated using the “pheatmap” R package. The volcano maps were constructed using the “ggplot2” R package.

### 2.5. Development of the Diagnostic Efficiency-Based Classifier Using Multiple Machine Learning Algorithms

Data from GSE26549, GSE85195, and GSE85514 were combined; a total of 197 samples, including 125 OL and 72 OSCC samples, of which 35 OL patients had progressed to OSCC, was used for further analysis. A 10-fold cross-validation was used to test the accuracy of the algorithm. The dataset was divided into ten parts, of which nine were used as training data, and one was used as validation data. Each trial generated a corresponding accuracy (or error rate). To obtain more accurate results, 10-fold cross-validation was performed ten times for each modeling. The mean value was calculated to estimate the accuracy of the algorithm.

To accurately predict the status of the progression from OL to OSCC, 10-fold cross-validation glmnet (cv. glmnet), glmnet, regression partition trees (rpart), k-nearest neighbor (KNN), linear discriminant analysis (LDA), logistic regression (log_reg), multinomial logit model (MNL), naïve Bayes, neural network (NNet), random forest, support vector machines (SVM), and extreme gradient boosting (XGBoost) analyses were used to build the models. A receiver operating curve (ROC) was used to evaluate the effect of the model on the validation set using the “mlr3” R package [23]. The area under the curve (AUC) was calculated using the “pROC” R package [24] to measure the prediction accuracy.

### 2.6. Enrichment Analysis

Gene Ontology (GO) enrichment analysis [25] is commonly used for performing large-scale functional enrichment analysis, including biological processes (BPs), molecular functions (MFs), and cellular components (CCs). The Kyoto Encyclopedia of Genes and Genomes (KEGG) pathway enrichment analysis [26] stores information about genomes, biological pathways, diseases, and drugs. GO and KEGG pathway enrichment analyses of differentially expressed genes were performed using the “clusterProfiler” R package [27]. The cut-off value of false discovery rate (FDR) < 0.05 was considered statistically enriched.

Gene set enrichment analysis (GSEA) was performed to investigate the differences in the biological processes between the groups [28] based on the gene expression profiling data from the GSE85195 dataset. GSEA is a computational method to analyze if a gene set is statistically different between two biological conditions. GSEA estimates changes in the pathways and biological processes in samples of datasets. The gene sets “c2.cp.kegg.v6.2.-symbols” and “c2.all.v7.2.symbols” were retrieved from the Molecular Signatures Database (MSigDB) [29] for GSEA analysis. FDR < 0.05 was considered significantly enriched.

### 2.7. Cluster Analysis

Consensus clustering determines the number and members of possible clusters in a dataset (microarray gene expression). “ConsensusClusterPlus” R package [30] was used to study the expression of model genes that consistently cluster into a merged dataset to help distinguish the different subtypes of OL progressing to OSCC. For this analysis, the number of clusters was set between 2 and 10, and the analysis for 80% of the total sample was repeated 100 times (clusterAlg = “pam”; distance = “Euclidean”).

### 2.8. Immune Infiltration Analysis

The tumor microenvironment (TME) is a comprehensive, integrated system, mainly composed of tumor tissue, surrounding immune and inflammatory cells, tumor-related fibroblasts, stromal tissue, various cytokines, and chemokines. The analysis of immune cell infiltration in cancer tissues plays an important role in understanding the pathogenesis of disease and predicting prognosis.

Cell-type Identification by Estimating Relative Subsets of RNA Transcripts CIBERSORTx [31] is an analytical tool to impute gene expression profiles and estimate the abundances of cell types in a mixed cell population using gene expression data. The expression matrix data of the training and validation sets were uploaded to CIBERSORTx separately, combined with the LM22 eigenene matrix. The immune cell infiltration matrix was created using filtered samples with an output of *p* < 0.05. The bar graphs show the distribution of 22 immune cell infiltrates in each sample. The single cell dataset GSE139324 retrieved from the Tumor Immune Single-Cell Hub (TISCH) database (http://tisch.comp-genomics.org/) (accessed on 8 January 2021) was used to verify the effect of gene expression on immune cell infiltration by analyzing the model gene expression in immune cells in the immune microenvironment [32].

### 2.9. RNA Isolation and Quantitative Real-Time Polymerase Chain Reaction (RT-qPCR)

Total RNA was extracted using the TRIzol reagent (Invitrogen, Carlsbad, CA, USA). Reverse transcription was performed using a PrimeScript RT reagent kit (Takara, Maebashi, Japan). RT-qPCR was performed using SYBR Green qPCR Master Mix (Takara, Maebashi, Japan) on a StepOnePlus qPCR machine (Thermo Fisher, Waltham, MA, USA). β-actin was used as an internal control. The primer sequences are shown in the Supplementary Table S2.

### 2.10. Prognostic Analysis

A nomogram characterizes multiple variables in the multivariate regression model, and the total score is calculated to predict the probability of events. The model genes were used as independent variables to assess the ability of each gene to predict patient outcomes. Simultaneously, the actual results were evaluated using the actual and predicted probabilities of the model in the calibration diagram.

The risk score formula was also calculated by optimizing the expression of genes, and the correlation was estimated by Cox regression coefficients. As described previously [33], the risk scores were calculated using a combination of the expression of chosen genes weighted by their respective Cox regression coefficients using the following formula:

“Risk score” = Σ (regression coefficient) × (expression value of each prognostic gene).

Patients were divided into high-score and low-score groups based on the specified median score. Kaplan–Meier (KM) survival analysis and the log-rank test were performed to analyze the overall survival (OS) rate on the TCGA-OSCC dataset. The Cox regression and KM analyses were performed via the “survival” R package.

### 2.11. Statistical Analysis

All calculations and statistical analyses were performed using R package software (version 4.0.2). The correlations between two continuous variables were calculated using Pearson’s correlation coefficients. The chi-squared test was used to compare categorical variables, and the continuous variables were compared using the Wilcoxon rank-sum test or Student’s t-test. One-way analysis of variance (ANOVA) was used to compare the three groups of continuous variables; *p* < 0.05 was considered statistically significant unless otherwise stated.

## 3. Results

### 3.1. Gene Expression Modules Associated with OL

**Figure 1:**
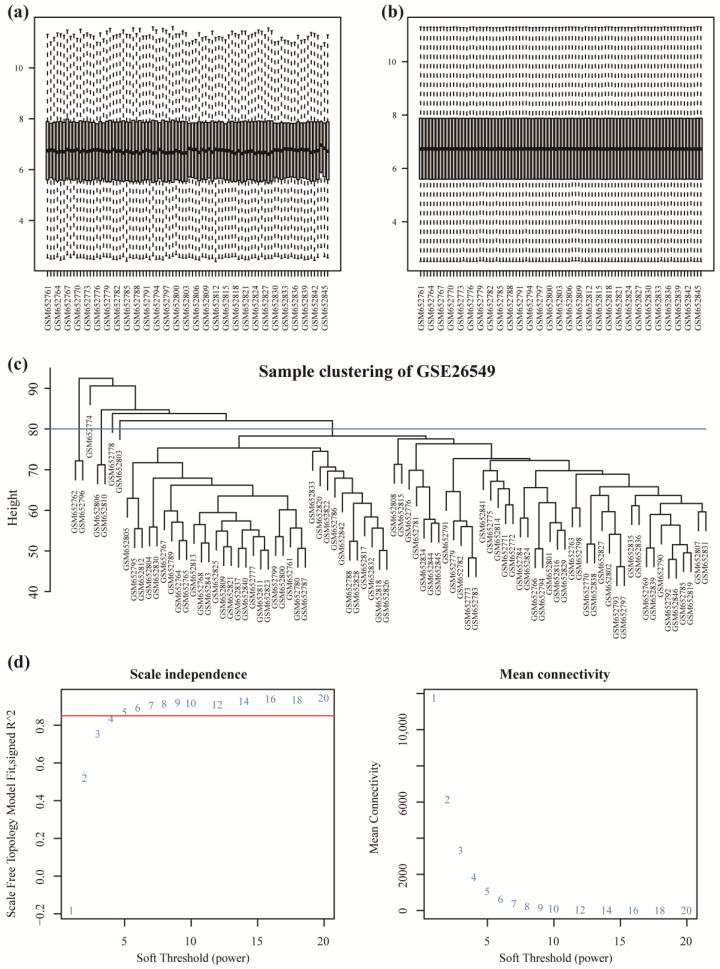
WGCNA of gene modules associated with OL. (**a**) Data distribution before the correction. (**b**) Data distribution after the correction. (**c**) Sample clustering tree. (**d**) The soft threshold value was calculated using the pickSoftTreshold function.

The gene expression matrix and clinical information of the GSE26549 dataset were retrieved from the GEO database. The dataset was comprised of 86 OL samples, of which 54 were hyperplasia and 32 were dysplasia. The “limma” R package was used for the standardized correction, and the results were obtained before (Figure 1a) and after the correction (Figure 1b). The outlier samples were removed based on the results of sample clustering, and the remaining 79 samples were clustered (Figure 1c). WGCNA constructed unsupervised clustering networks for identifying different gene sets. The soft threshold value was calculated based on the pickSoftTreshold function. The most critical parameter of the soft threshold value power was set to 5 (Figure 1d) to ensure the overall connectivity of the co-expression module.

**Figure 2:**
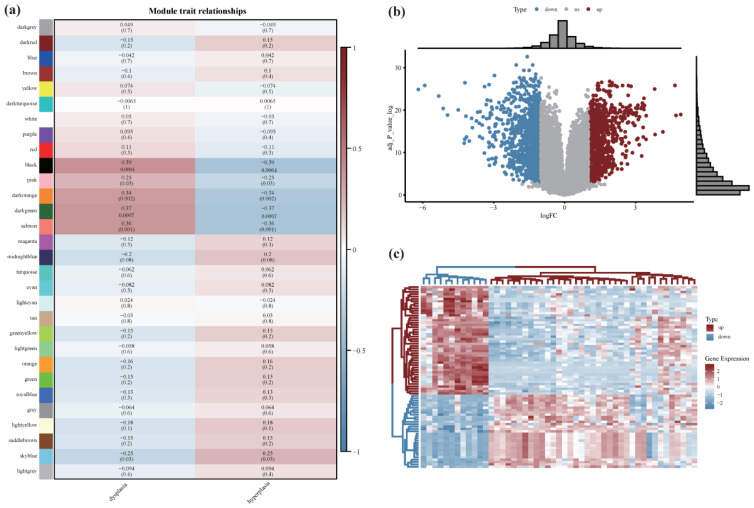
Differential expression analysis between OL and OSCC: (**a**) 30 co-expression modules were constructed and displayed in different colors; (**b**) the abscissa is log2 fold change (logFC), the ordinate is log (adj *p*-value), red nodes are up-regulated differentially expressed genes (DEGs), while blue nodes are down-regulated DEGs, and gray nodes are not significantly DEGs. (**c**) The expression trend of DEGs was represented using a heat map. Red color indicates up-regulation, and blue indicates down-regulation.

Further, thirty co-expression modules were constructed and displayed in different colors (Figure 2a). A significant positive correlation was observed between the black (r = 0.39, *p* = 7 × 10^−4^), pink (r = 0.25, *p* = 0.03), dark orange (r = 0.34, *p* = 0.002), dark green (r = 0.37, *p* = 7 × 10^−4^), and salmon (r = 0.36, *p* = 0.001) module genes and dysplasia leukoplakia. In total, 488 genes from the most strongly correlated black module were used for the subsequent analysis.

### 3.2. Differential Expression Analysis between OL and OSCC

The GSE85195 dataset is comprised of OL, OSCC, and normal tissue mucosa samples. The normal samples were discarded, and OL and OSCC samples were grouped for subsequent analysis.

The raw data of GSE85195 was retrieved from the GEO for probe filtering, and a box plot was constructed to analyze the data distribution (Figure S1). The differential analysis was performed using the “limma” R package to screen for genes with a |logFC| > 1 and a corrected *p* < 0.05 (Figure 2b). Compared to OL samples, 931 genes were upregulated, and 1144 genes were downregulated in OSCC samples. As shown in Figure 2c, a heatmap was constructed based on the partial genes to display the expression trend. The results revealed significant differences in differentially expressed genes. The differentially expressed genes were separated and intersected with the 448 genes in the black module previously associated with OL (Figure S2). The up-regulated gene intersection had 27 genes, and the down-regulated gene intersection had one gene (Supplementary Table S3).

### 3.3. Development and Verification of the Diagnostic-Efficiency-Based Classifier Using Multiple Machine Learning Algorithms

**Figure 3:**
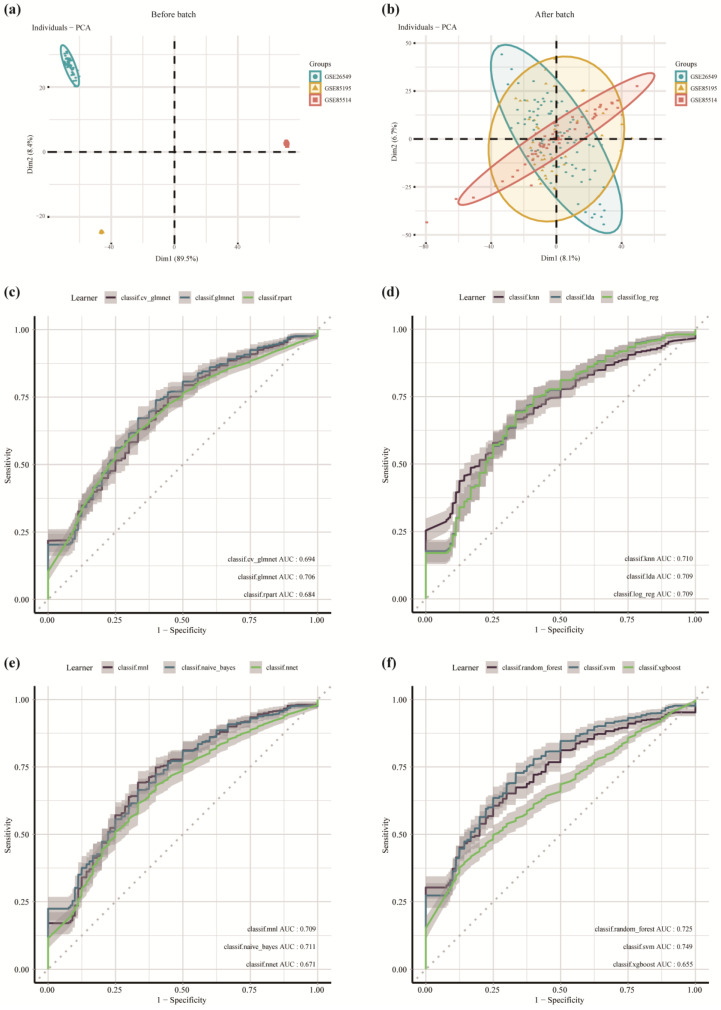
Development and verification of the diagnostic efficiency-based classifier using multiple machine learning algorithms. (**a**) Principal component analysis (PCA) before removing the batch effects. (**b**) PCA after removing the batch effects. (**c**) Machine learning algorithms: cv. glmnet (10-fold cross-validation glmnet), glmnet, rpart (regression partition trees). (**d**) Machine learning algorithms: KNN (k-nearest neighbor), LDA (linear discriminant analysis), log_reg (logistic regression). (**e**) MNL (multinomial logit model), naïve Bayes, NNet (neural network). (**f**) random forest, SVM (support vector machines), XGBoost (extreme gradient boosting).

Twelve machine learning algorithms were used to determine if these genes had diagnostic efficacy to predict the progression from OL to OSCC. To expand the sample size of the constructed model, the GSE26549, GSE85195, and GSE85514 datasets were merged, and all OL and OSCC samples were retained. The intersection of the merged genes with the 27 up-regulated genes revealed that seven genes, including *DHX9, BCL2L12, RAD51, MELK, CDC6, ANLN,* and *KIF4A,* were considered risk factors for OL progression to OSCC. The batch effect was removed after merging the datasets. Principal component analysis showed the result before (Figure 3a) and after removing the batch effect (Figure 3b). The results revealed that the three datasets could mix well after the batch effect was removed. We analyzed and visualized the expression of the seven genes in the merged datasets (Figure S3). These genes were highly expressed in OSCC compared to OL samples (*p* < 0.05).

The model was constructed using twelve machine learning algorithms. The ROC curve was used to calculate the AUC for evaluating the model efficiency (Figure 3c–f). The model efficiency of the twelve machine learning algorithms is shown in Table 1. The table shows the AUC of the training set and the test set, respectively, representing the ability of the models to predict the risk of carcinogenesis. There were differences between the constructed models. The SVM algorithm constructed the models with the highest performance, with a test set AUC = 0.748687. The performance of the model constructed by the naïve Bayes algorithm was similar in both the training and the validation sets, with the train set AUC = 0.7281341 and the test set AUC = 0.7108963, indicating that the model was a good fit during the training process. However, the performance of the model constructed by the random forest algorithm was different in the training and the validation sets, with the training set AUC = 0.9985012 and the test set AUC = 0.7248809. This suggests that the model was overfitted during the training process and should be corrected by validation. The AUC > 0.65 for twelve models in the validation set. This indicates that the models had good efficiency, and the genes included in these models could differentiate between OL and OSCC. These results suggest that models had diagnostic efficacy and could be used in the clinical setting to predict the susceptibility of OL progressing to OSCC.

### 3.4. Enrichment Analysis

**Figure 4:**
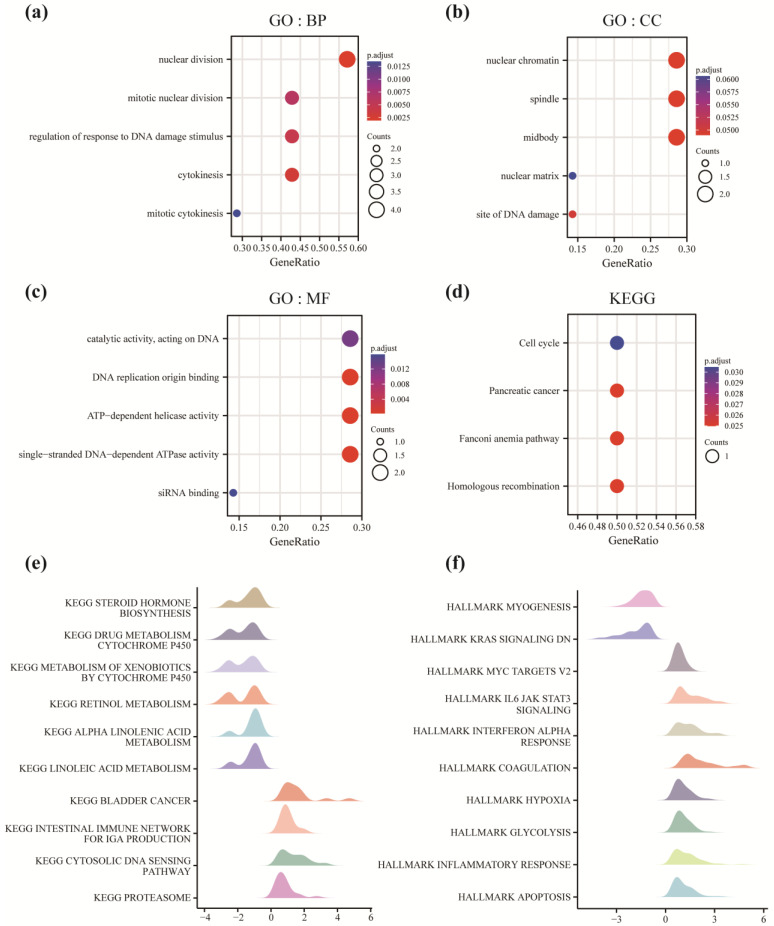
GO, KEGG, and GSEA. (**a**–**c**) Gene ontology (GO) enrichment analysis terms include Biological Process (BP), Cellular Component (CC), and Molecular Function (MF). The abscissa is the gene ratio, and the ordinate is the GO terms. The node size indicates the number of genes enriched, and the node color indicates −log10 (*p*-value). (**d**) For the Kyoto Encyclopedia of Genes and Genomes (KEGG) pathway enrichment analysis, the abscissa is the gene ratio, and the ordinate is the pathway name. The node size indicates the number of genes enriched in the pathway, and the node color indicates −log10 (*p*-value). (**e**) Gene-set enrichment analysis (GSEA) for OL and OSCC. The results are visualized in the form of mountain maps. The abscissa is the gene ratio, and the ordinate is KEGG. The color indicates the *p*-value. (**f**) GSEA for OL and OSCC samples. The results are visualized in the form of mountain maps. The abscissa is the gene ratio, and the ordinate is HALLMARK. The color indicates the *p*-value.

Next, the underlying mechanisms associated with the progression from OL to OSCC were investigated. GO and KEGG pathway enrichment analyses were performed using the seven genes to predict the functions and signaling pathways associated with the progression from OL to OSCC (Supplementary Table S4). The BP term (Figure 4a) enriched were nuclear division, mitotic nuclear division, regulation of response to DNA damage stimulus, cytokinesis, and mitotic cytokinesis. The CC term (Figure 4b) enriched by these genes were nuclear chromatin, spindle, midbody, nuclear matrix, and site of DNA damage. As shown in Figure 4c, the MF term enriched were catalytic activity, acting on DNA, DNA replication origin binding, ATP-dependent helicase activity, single-stranded DNA-dependent ATPase activity, and siRNA binding. KEGG pathway enrichment analysis revealed that the pathways enriched by these genes were the cell cycle, pancreatic cancer, Fanconi anemia, and homologous recombination pathways (Figure 4d). The enrichment analysis showed that the key genes involved in the progression from OL to OSCC mainly affected processes such as cell division, including nuclear division, DNA damage repair, and ATP-dependent enzyme activity. Further, the genes were associated with signaling pathways such as the cell cycle, which alters cell proliferation and promotes the transformation of normal cells to continuously dividing cells.

GSEA was performed to identify the differences in the biological processes between OL and OSCC samples (Supplementary Table S5). The results revealed differences in the pathways between the groups as follows (Figure 4e): STEROID_HORMONE_BIOSYNTHESIS, DRUG_METABOLISM_CYTOCHROME_P450, METABOLISM_OF_XENOBIOTICS_BY_CYTOCHROME_P450, RETINOL_METABOLISM, ALPHA_LINOLENIC_ACID_METABOLISM, LINOLEIC_ACID_METABOLISM, BLADDER_CANCER, INTESTINAL_IMMUNE_NETWORK_FOR_IGA_PRODUCTION, and CYTOSOLIC_DNA_SENSING_PATHWAY, PROTEASOME. The differences in the functions between the two groups were as follows (Figure 4f): MYOGENESIS, KRAS_SIGNALING_DN, MYC_TARGETS_V2, IL6_JAK_STAT3_SIGNALING, INTERFERON_ALPHA_RESPONSE, COAGULATION, HYPOXIA, GLYCOLYSIS, INFLAMMATORY_RESPONSE, and APOPTOSIS. Based on these results, we hypothesized that during the progression from OL to OSCC, there is a decrease in the expression of genes related to fat metabolism and an increase in the expression of genes related to glycolysis, immune infiltration, inflammatory response, and transcription of MYC. Together these results indicate an increased risk of tumorigenesis and oncogenesis.

### 3.5. Subtype Analysis of the Model Genes

**Figure 5:**
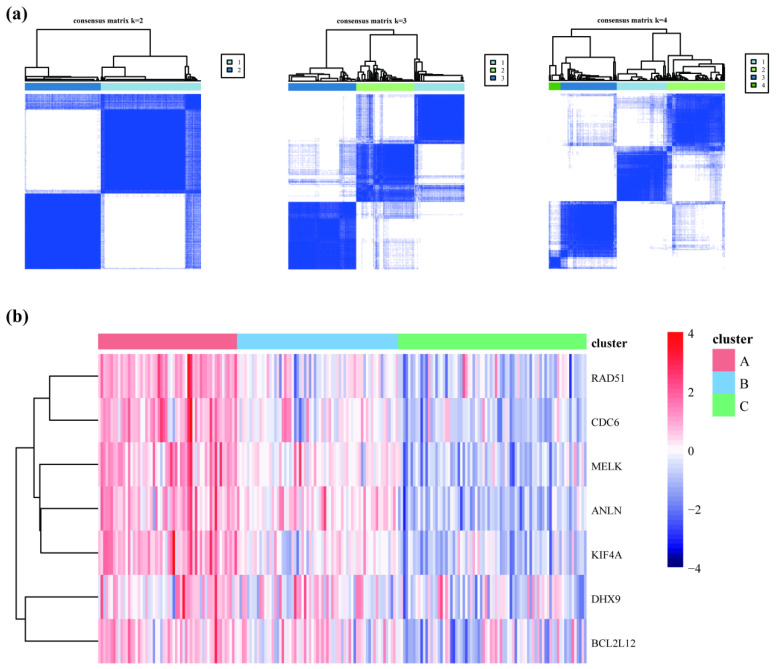
Subtype analysis of the model genes. (**a**) The consensus clustering analysis was performed, and the number of categories was 2, 3, and 4, respectively. (**b**) Heatmaps were constructed by grouping the subtypes and represent the expression of the model genes. Red indicates upregulation, and blue indicates downregulation.

The “ConsensusClusterPlus” R package was used to study the effects of the model genes on the progression from OL to OSCC by consistently clustering the pooled dataset using the model genes described above (Figure 5a). The results showed that the sample classification performed well when the typing number parameter was 3. The combined samples were classified into 56 samples in type A, 65 samples in type B, and 76 samples in type C. To determine the significance of each subtype, a heatmap was constructed based on the model genes classified into subtypes (Figure 5b). The results showed a significant difference in the model gene expression trend. The samples clustered in subtype A were the high-risk group for progression from OL to OSCC, samples clustered in subtype B were the medium-risk group for progression from OL to OSCC, and samples clustered in subtype C were the low-risk group for progression from OL to OSCC. The results of the three groups were retained for subsequent analysis.

### 3.6. Model Genes Affect Immune Cell Infiltration

**Figure 6:**
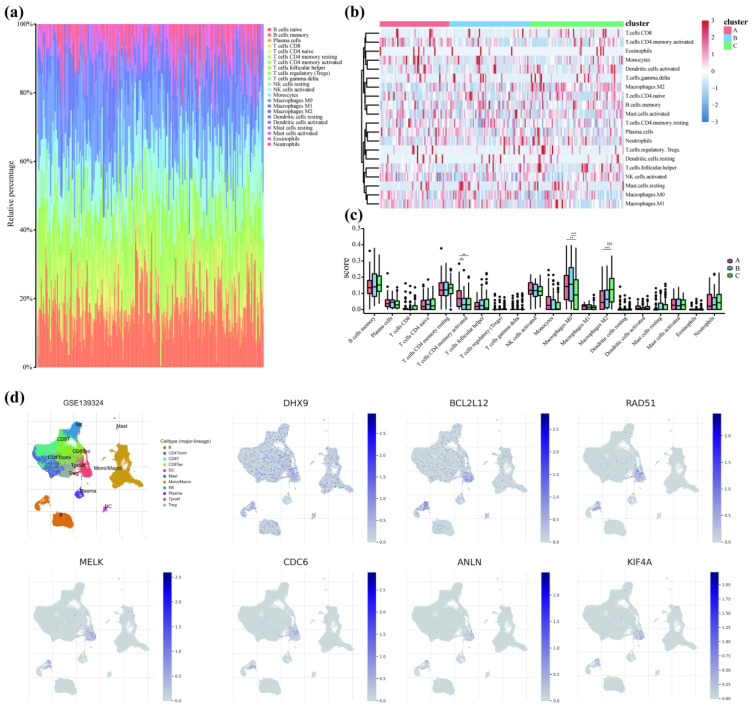
Model genes affecting immune cell infiltration. (**a**) Bar plot of immune cell correlations was analyzed using CIBERSORTx. (**b**) Heatmap constructed based on subtype groups shows the infiltration of different immune cells in three subtypes. Red indicates upregulation, and blue indicates downregulation. **(c)** Group comparison plot of various immune cells grouped according to subtypes (** *p* < 0.01; *** *p* < 0.001). (**d**) The expression of model genes in different immune cells using single-cell from GSE139324. B: B cells, CD4Tconv: conventional CD4 T cells, CD8T: CD8 T cells, CD8Tex: exhausted CD8 T cells, DC: dendritic cells, Mast: mast cells, Mono/Macro: monocytes or macrophages, NK: natural killer cells, Plasma: plasma cells, Tprolif: proliferating T cells, Treg: regulatory T cells.

CIBERSORTx was used to study the effects of model genes on the infiltration of immune cells by calculating the degree of 22 immune cell infiltration, such as naïve B cells, memory B cells, plasma cells, CD8 T cells, naïve CD4 T cells, memory resting CD4 T cells, activated CD4 T cell, follicular helper T cells, regulatory T cells, gamma delta T cells, resting NK cells, activated NK cells, monocyte, M0 macrophages, M1 macrophages, M2 macrophages, resting dendritic cells, activated dendritic cells, resting mast cells, activated mast cells, eosinophils, and neutrophils (Figure 6a). The differences in immune cell infiltration between the different subtypes were analyzed. A heatmap of correlation between various immune cells was constructed. The low abundance naïve B cells and resting NK cells were excluded, and the remaining cells were included in the analysis (Figure 6b). The results showed a significant difference in the infiltration of CD4 T cells and macrophages between the different subtypes. A group comparison plot was drawn to analyze the differences in immune cell infiltration between the different subtypes (Figure 6c). A significant difference in memory-activated CD4 T cells, M0 macrophages, and M2 macrophages was observed between the groups. Increased CD4 T cell infiltration and M0 to M2 macrophage polarization were observed in samples with an increased risk of progression from OL to OSCC.

To analyze the effect of model genes on immune cells, the expression of the model genes in immune cells was analyzed using single-cell data from the GSE139324 dataset (Figure 6d). The results revealed an increase in expression of all seven model genes in proliferating T cells. An increase in the expression of *DHX9* and *BCL2L12* in macrophages was observed, which could likely be involved in macrophage polarization.

### 3.7. Prognostic Analysis

**Figure 7:**
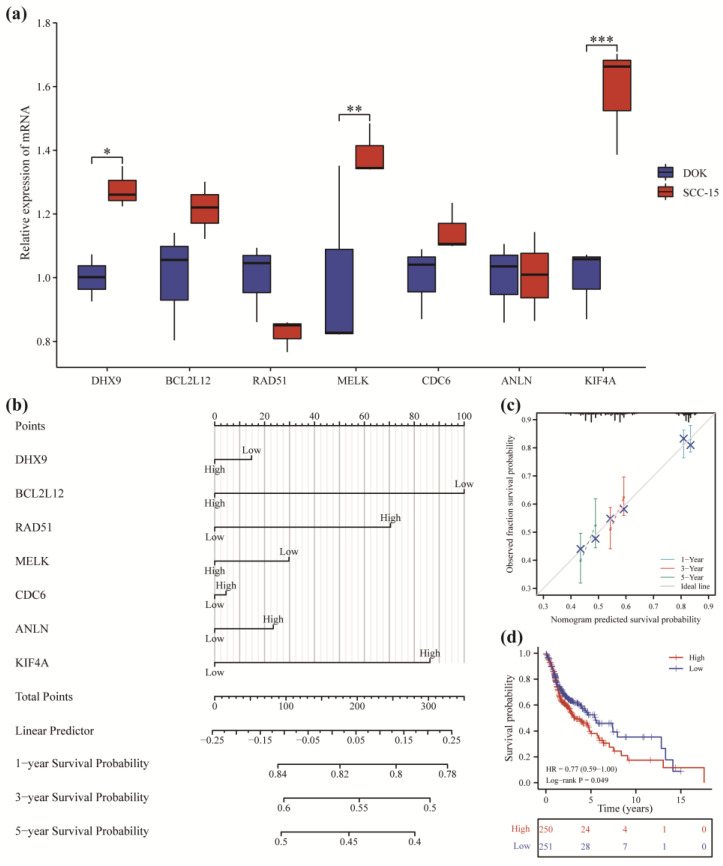
Prognostic analysis of the model genes. (**a**) Relative mRNA expression differences in DOK cells and SCC-15 cells. The experiments were repeated in three independent experiments (* *p* < 0.05; ** *p* < 0.01; *** *p* < 0.001). (**b**) Nomogram of prognostic correlation of model genes. (**c**) Calibration diagram of 1, 3, and 5 years. (**d**) Kaplan–Meier survival curve was plotted based on the risk score to analyze the overall survival.

TCGA-OSCC paired data were used to study the differential expression of model genes in adjacent normal and tumor tissues. The results revealed a significant increase (*p* < 0.001) in the expression of all seven genes in tumor tissues compared to normal tissues (Figure S4). Further, we detected the mRNA expression of seven genes in oral mucosal precancerous cells and oral squamous cell carcinoma cells (Figure 7a). The results showed that the mRNA of DHX9, MELK, and KIF4A in oral squamous cell carcinoma were significantly higher than those in precancerous lesions, indicating that the transcription level of these three genes was increased during carcinogenesis, and the other four genes may play roles in promoting tumorigenesis through post-translational modification.

The effects of the model genes on the prognosis of OSCC patients was then analyzed based on TCGA-OSCC clinical data. The nomogram showed that each gene contributed differently to predicting the patient prognosis (Figure 7b), of which BCL2L12, KIF4A, and RAD51 could better predict the overall survival rate of the patients. The calibration diagram (Figure 7c) showed that the prediction results were close to the ideal, indicating good prediction accuracy. The risk score formula was then calculated by Cox regression:

(1) Risk score =DHX9×−0.021973279+BCL2L12×−0.148809986+RAD51×0.104871934+MELK×−0.04432069+CDC6×0.006716715+ANLN×0.03493475+KIF4A×0.128401155.

The dataset was divided into a high-risk and low-risk group based on the median score, and the KM survival curve was plotted (Figure 7d). The results revealed that the overall survival rate of the high-risk group was lower compared to the low-risk group (HR = 0.77, *p* = 0.049). The results showed that the risk score could better predict survival at 3–10 years.

## 4. Discussion

The diagnosis of the progression from OL to OSCC is still dependent on the interpretation of the pathologists and lacks standardization. Therefore, it is necessary to identify key genes associated with the progression from OL to OSCC for effective diagnosis. In this study, we used OL samples that did not progress to OSCC, OL samples that progressed to OSCC, and OSCC samples. Based on the analysis, seven key genes, including *DHX9, BCL2L12, RAD51, MELK, CDC6, ANLN,* and *KIF4A*, were identified, which were associated with OL progression to OSCC. The diagnostic efficacy was verified using twelve machine learning algorithms. The results revealed that these seven genes could effectively predict the progression from OL to OSCC and may also be associated with the prognosis of OSCC patients.

Of the seven genes identified, previous studies have shown the involvement of only *CDC6* [34] in the progression from OL to OSCC. A gradual increase in *CDC6* was observed in the normal mucosa, OL, and OSCC tissues, which is consistent with our findings. Yuyang Li [35] reported that the *RAD51* expression was higher in OSCC compared to normal mucosal tissues. Yasuyuki Minakawa [36] and Yun Zhang [37] showed that *KIF4A* promotes the proliferation of OSCC cell lines. A study by Bo Li [38] reported that *MELK* promotes the proliferation and migration of OSCC cells. Further, high *MELK* expression was correlated with a poor prognosis in OSCC patients. However, these studies did not analyze the expression of these genes in OL; hence, these studies failed to shed light on the role of these genes in the malignant transformation of leukoplakia. To the best of our knowledge, our study is the first to use multiple machine learning algorithms to predict the diagnostic efficacy of key genes progressing from OL to OSCC. Twelve machine learning algorithms were used to construct models, and the results revealed that all the models have good diagnostic efficacy. This indicates that the expression of these genes could accurately predict the progression from OL to OSCC. The performance of these models was compared, and the results revealed that the model constructed by the naïve Bayes algorithm performed consistently in both the training and the validation sets. Further, the model constructed by the random forest algorithm showed a significant difference between the training and the validation sets. These results suggest that the model constructed using the naïve Bayes algorithm performed better than other diagnostic models constructed in this study. Further, the model constructed using a random forest algorithm needs to be more rigorous.

In addition to the effect of these genes on tumor cells, we next analyzed if these genes could affect the immune microenvironment, which plays an important role in the neoplastic transformation of normal cells [39]. The TME mainly includes cancer cells, immune cells, and stromal cells. The malignant tumor cells alter immune cell infiltration, which plays an important role in tumor development [40]. Hence, subtype analysis was conducted to divide the samples into three subtypes: high, medium, and low-risk of progression from OL to OSCC. Next, the differences in immune cell infiltration were analyzed among the three subtypes to explore the changes in immune cell infiltration during OL to OSCC progression. Previous studies have shown that M2 macrophages promote tumor progression [41,42,43]. Our results revealed that in addition to an increase in M2 macrophage infiltration, a significant increase in infiltration of CD4 T cells was observed during the progression from OL to OSCC. CD4 T cells infiltrating the TME may help tumor tissues escape immune surveillance. Our results provide the theoretical basis for cancer therapies targeting tumor-associated macrophages and T cells; however, additional research is still required to support the hypothesis.

The expression of key genes in immune cells affects immune cell infiltration; hence, the enrichment of seven genes in various immune cells was analyzed using single-cell data. The results revealed significant enrichment of all the genes in proliferating T cells. Further, high expression levels of *DHX9* and *BCL2L12* were observed in macrophages. These results indicate that these genes may alter the tumor immune microenvironment by influencing the differentiation and immune cell infiltration in leukoplakia lesions, thereby affecting the progression from OL to OSCC. In this study, the number of patients who reported progression from OL to OSCC was low, and there was no patient follow-up. Hence, determining the diagnostic efficacy and underlying molecular mechanisms of these genes requires in-depth experimental validation and exploration.

Various studies have shown that the expression of these seven genes could be used as prognostic markers, predicting survival in patients with cancers, including lung cancer [44,45,46], glioma [47,48], breast cancer [49,50,51,52], esophageal squamous cell carcinoma [53], hepatocellular carcinoma [54,55,56,57,58], gastric cancer [59], colorectal cancer [60,61,62,63,64], and renal cell carcinoma [65,66]. Very few studies have analyzed the prognostic efficiency of these genes in OSCC. Therefore, in this study, we investigated the impact of these genes on the prognosis of OSCC patients using TCGA-OSCC data. To the best of our knowledge, our study is the first to include these seven genes to evaluate the overall prognosis of OSCC patients using the Cox regression model. Although these seven genes were highly expressed in OSCC compared to OL, the efficacy in predicting the prognosis in tumor patients was not consistent. The high expression of *RAD51, CDC6, ANLN*, and *KIF4A* could reduce patient survival, while the high expression of *DHX9, BCL2L12*, and *MELK* could increase the probability of patient survival. This suggested that the molecular mechanisms played by these three genes during carcinogenesis and tumor progression may be different, and more studies are needed to confirm this conjecture.

In conclusion, our study screened and verified key genes involved in the progression from OL to OSCC and analyzed their diagnostic efficiency. Our results suggest that *DHX9, BCL2L12, RAD51, MELK, CDC6, ANLN,* and *KIF4A* expression could be important diagnostic indicators for the progression from OL to OSCC. These genes could potentially be used for early diagnosis and intervention for patients with high risk of OSCC. Preliminarily analysis was performed to study the underlying mechanisms of these genes associated with tumor initiation and development. However, additional studies are needed to validate and explore the molecular mechanisms underlying the progression from OL to OSCC.

## 5. Conclusions

The study screened for seven genes that may play key roles in the progression from OL to OSCC, including *DHX9, BCL2L12, RAD51, MELK, CDC6, ANLN*, and *KIF4A*. The results of the twelve machine learning models showed that these seven genes have great diagnostic efficacy, which may improve the diagnostic accuracy of the transformation from OL to OSCC and help in early prevention and intervention to reduce the incidence of OSCC.

## Figures and Tables

**Table 1:** The efficiency of twelve models based on multiple machine learning algorithms.

Learner_Id	AUC_Train	AUC_Test
classif.cv_glmnet	0.7163359	0.6935978
classif.glmnet	0.7451895	0.7058361
classif.rpart	0.8395653	0.6842594
classif.knn	0.962321	0.7103622
classif.lda	0.7493833	0.709229
classif.log_reg	0.749681	0.7086145
classif.mnl	0.7496823	0.7086145
classif.naïve_Bayes	0.7281341	0.7108963
classif.nnet	0.7839936	0.6708493
classif.random_forest	0.9985012	0.7248809
classif.svm	0.8655543	0.748687
classif.xgboost	0.9261073	0.6546356

## Data Availability

Raw data are available at the TCGA database (https://portal.gdc.cancer.gov/) (accessed on 28 September 2022) and the GEO database: GSE26549 (https://www.ncbi.nlm.nih.gov/geo/query/acc.cgi?acc=GSE26549) (accessed on 1 February 2011), GSE85195 (https://www.ncbi.nlm.nih.gov/geo/query/acc.cgi?acc=GSE85195) (accessed on 26 April 2017), and GSE85514 (https://www.ncbi.nlm.nih.gov/geo/query/acc.cgi?acc=GSE85514) (accessed on 26 April 2017).

## Supplementary Materials

The following supporting information can be downloaded at: https://www.mdpi.com/article/10.3390/cancers14235808/s1, Figure S1: Box plot was drawn to view the data distribution in GSE85195; Figure S2: Venn diagram of the intersection of WGCNA genes with differential expression genes; Figure S3: Difference in gene expression in the oral leukoplakia (OL) and the oral squamous cell carcinoma (OSCC) using merged datasets (* *p* < 0.05; ** *p* < 0.01; *** *p* < 0.001; **** *p* < 0.0001; ns, *p* > 0.05); Figure S4: Difference in gene expression in the adjacent normal and the tumor tissues using TCGA-OSCC data (* *p* < 0.05; ** *p* < 0.01; *** *p* < 0.001; **** *p* < 0.0001; ns, *p* > 0.05); Table S1: Information on the GEO datasets; Table S2: Primer sequences; Table S3: Venn diagram result; Table S4: GO and KEGG pathway enrichment analyses; Table S5: Gene set enrichment analysis (GSEA).
