# Supplementary figures and images for: Screening for Biomarkers for Progression from Oral Leukoplakia to Oral Squamous Cell Carcinoma and Evaluation of Diagnostic Efficacy by Multiple Machine Learning Algorithms
Source: Cancers (Basel). 2022 Nov 25;14(23):5808. doi: 10.3390/cancers14235808 (PMC9738227; doi:10.3390/cancers14235808)

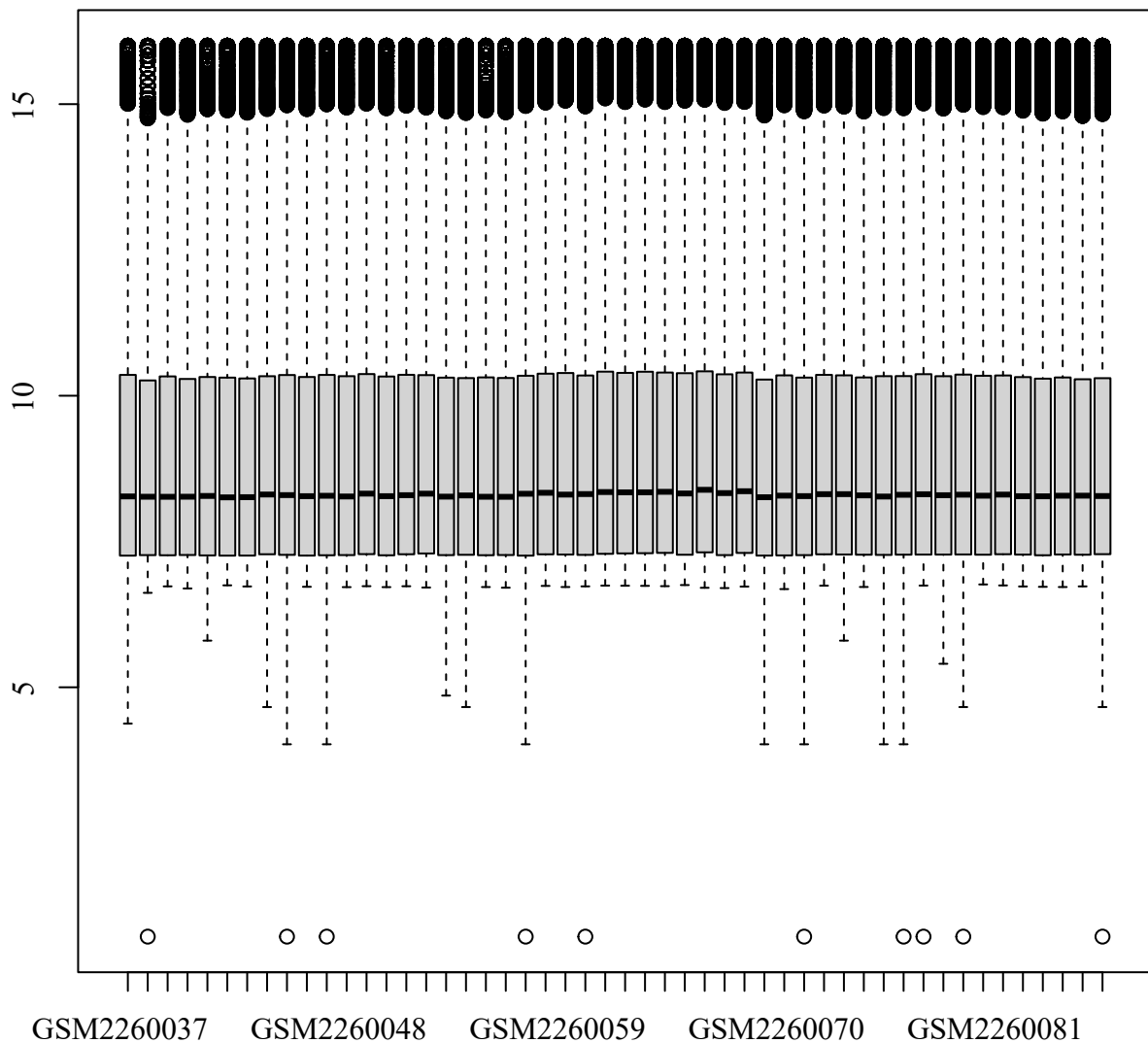

Supplement: Supplementary file 1 [file cancers-14-05808-s001.zip › Figure S1.pdf]

DEG\_Down\_Genes

WGCNA\_Hub\_Genes

DEG\_Up\_Genes

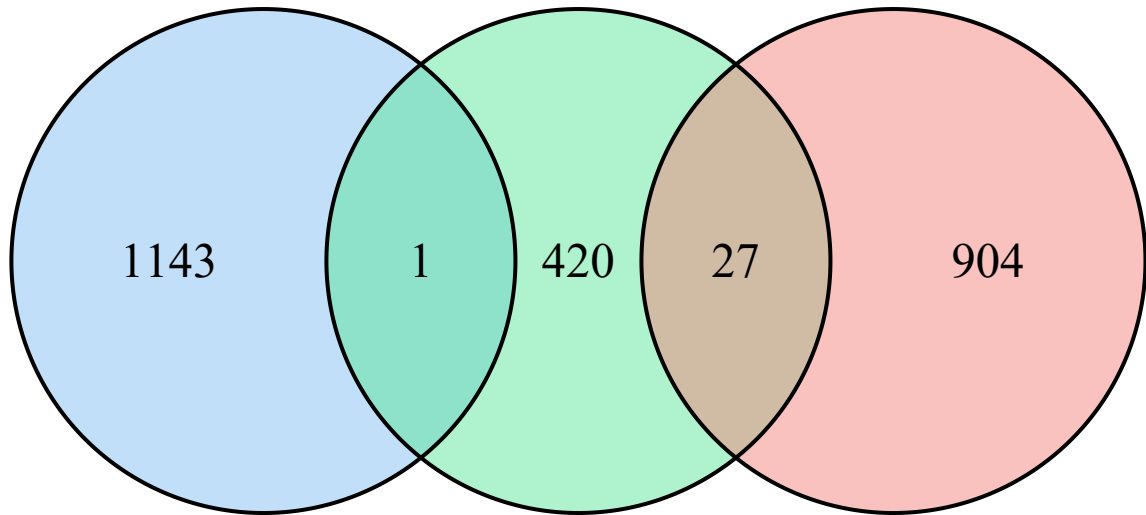

Supplement: Supplementary file 1 [file cancers-14-05808-s001.zip › Figure S2.pdf]

Merged Datasets

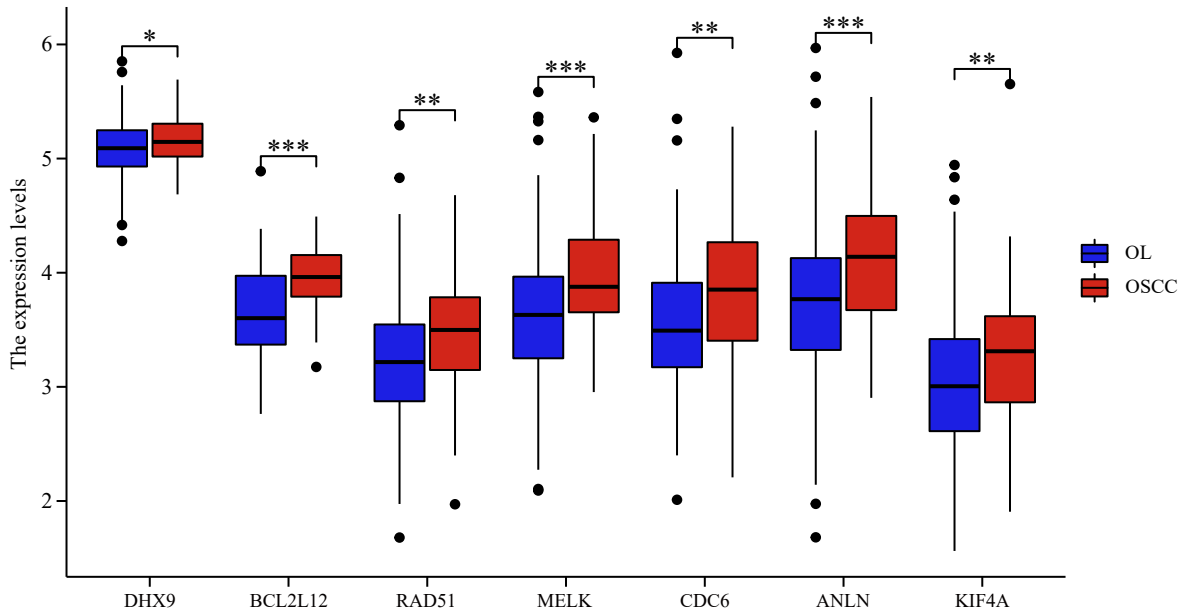

Supplement: Supplementary file 1 [file cancers-14-05808-s001.zip › Figure S3.pdf]

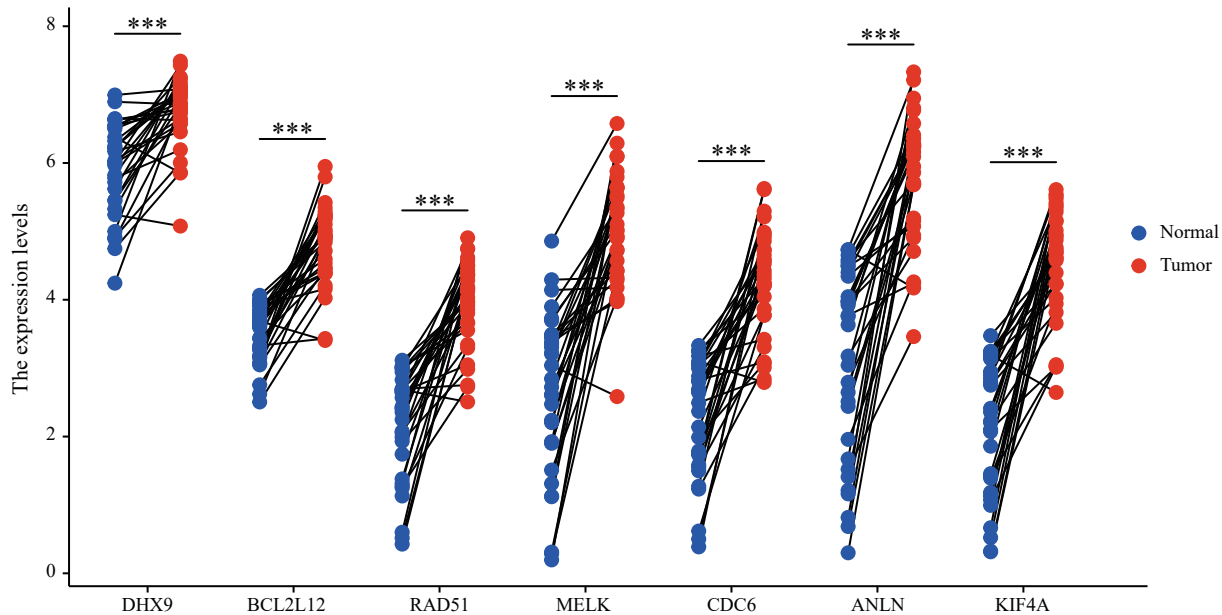

Supplement: Supplementary file 1 [file cancers-14-05808-s001.zip › Figure S4.pdf]
